# Autoeczematization: A Rare Complication of Contact Dermatitis Triggered by Topical Antibiotic Ointment

**DOI:** 10.7759/cureus.100040

**Published:** 2025-12-24

**Authors:** George Miura, Norihiro Yoshimoto, Suguru Kurosawa, Hiroyuki Nakamura

**Affiliations:** 1 Dermatology, Kushiro City General Hospital, Kushiro, JPN

**Keywords:** antibiotic ointment, autoeczematization, contact dermatitis, erythematous papules, id reaction

## Abstract

Topical antibiotic ointments are widely prescribed for infection prophylaxis after surgery and trauma, yet they are an important cause of allergic contact dermatitis. Autoeczematization, or id reaction, refers to secondary eczematous eruptions that arise at sites distant from a primary localized inflammatory focus.

We report a 42-year-old man who developed generalized exudative erythematous papules after application of Baramycin® (bacitracin and fradiomycin sulfate) ointment to an erosion on his right lower leg treated for cellulitis. Despite improvement of systemic inflammatory markers, well-demarcated erythema confined to the area of ointment and dressing use, accompanied by widespread papules on the trunk, face, and extremities, persisted. Delayed patch testing could have been performed after clinical improvement, but the patient was lost to follow-up, and the clinical course strongly suggested allergic contact dermatitis with secondary autoeczematization.

Discontinuation of Baramycin® and initiation of topical clobetasol propionate led to the gradual resolution of the primary lesion and rapid improvement of distant eruptions. This case underscores the need to consider autoeczematization in patients presenting with diffuse eczematous eruptions following the use of topical antibiotics.

## Introduction

Topical antibiotic ointments are widely used for infection prophylaxis after surgery and trauma, and are also applied in the treatment of superficial skin infections [1]. Although clinical evidence supporting their efficacy is somewhat limited, the ability to deliver high local concentrations of antibiotics at the site of infection and the reduced risk of systemic toxicity are considered major advantages [2]. However, topical ointments are among the most frequent causes of allergic contact dermatitis (second only to cosmetics) and thus represent important allergens [3].

Autoeczematization (an id reaction) often manifests as secondary eczematous eruptions distant from a primary localized inflammatory or infectious skin focus. Both allergic and irritant contact dermatitis can trigger immunologic responses that lead to widespread eczematous lesions [4]. We report a case of autoeczematization arising from contact dermatitis to a topical antibiotic ointment.

## Case presentation

**Figure 1:**
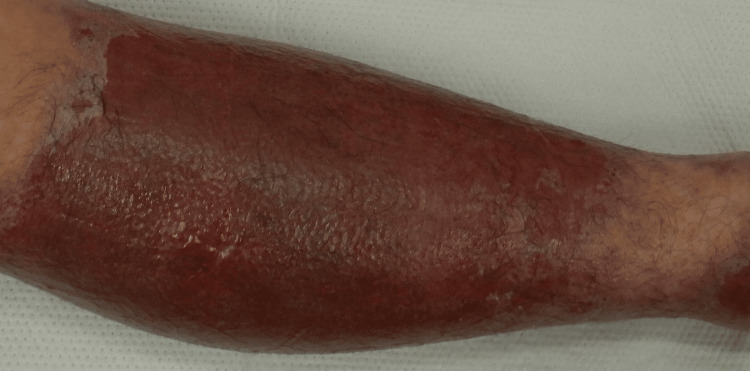
Well-demarcated erythema on the right lower leg. The lesion corresponds to the dressing area where topical Baramycin® ointment was applied.

**Figure 2:**
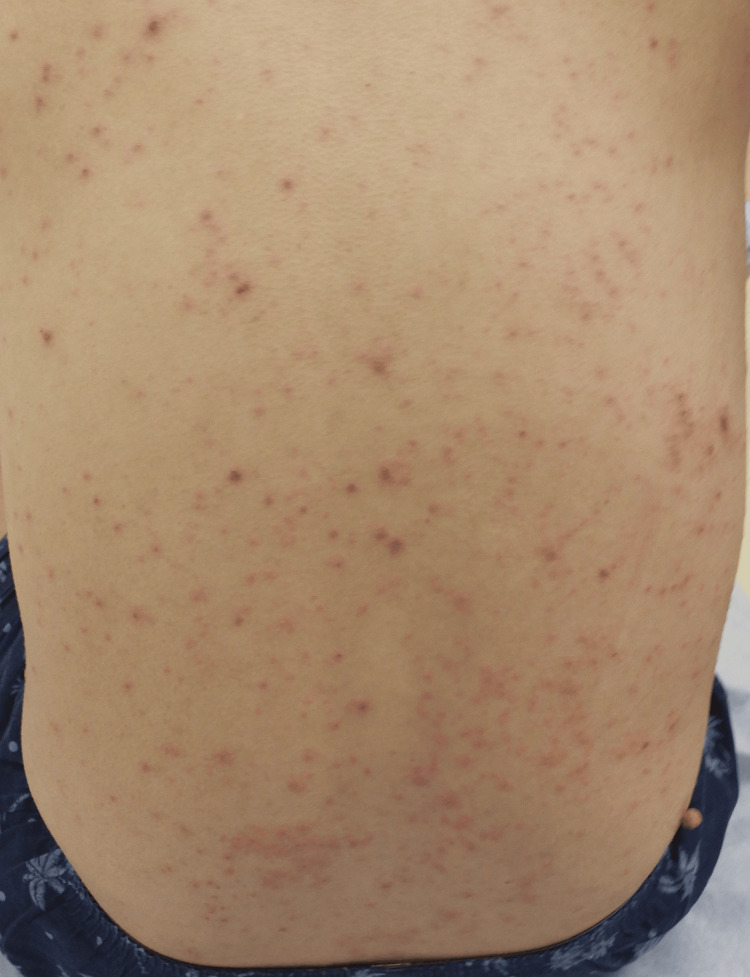
At the initial visit, the back shows widespread erythematous papules.

A 42-year-old man was referred to our department with persistent, exudative erythematous papules covering his trunk, limbs, and face (Figures 1, 2).

One month earlier, he had been admitted elsewhere for suspected cellulitis of the right lower leg that presented with erythema and swelling. Intravenous antibiotics were administered, and topical Baramycin® (a combination ointment of bacitracin and fradiomycin sulfate) ointment was applied to the erosion on the swollen area of the right lower leg for one month. However, his skin lesions gradually worsened, and one week before his presentation to our hospital, exudative erythematous papules appeared and spread from the right lower leg to the entire body. Despite the improvement of his systemic inflammatory markers, the diffuse eruptions persisted, prompting referral to our hospital.

Physical examination revealed well-demarcated erythema with small vesicles on the right lower leg, precisely corresponding to the area where the gauze dressing had been applied (Figure 1). In addition, erythematous papules were observed on the trunk, face, and extremities, accompanied by intense pruritus. Blood tests showed no remarkable abnormalities. Clinically, the eruption began as sharply demarcated erythema on the right lower leg and subsequently spread as erythema over the entire body. Patch testing was not performed at this stage because the eruptions were so extensive that no unaffected skin was available for testing. However, the distribution of the lesions and the clinical course strongly suggested generalized autoeczematization secondary to contact dermatitis induced by Baramycin® ointment.

**Figure 3:**
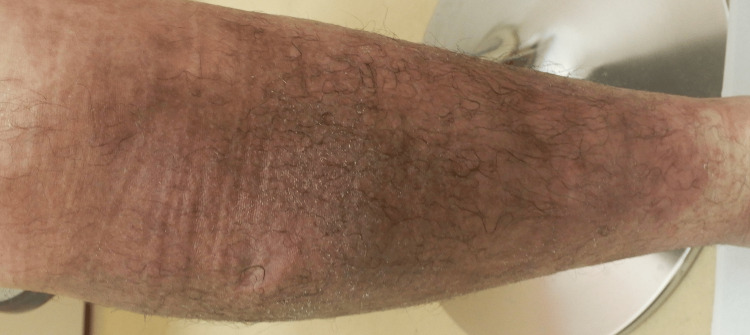
After three weeks of topical steroid therapy, the right lower leg shows significant improvement, with only residual hyperpigmentation remaining.

We initiated topical clobetasol propionate ointment therapy for the entire body, including the original right lower-leg lesion. Complete resolution of the right lower-leg lesion was achieved in approximately three weeks, leaving residual hyperpigmentation (Figure 3).

**Figure 4:**
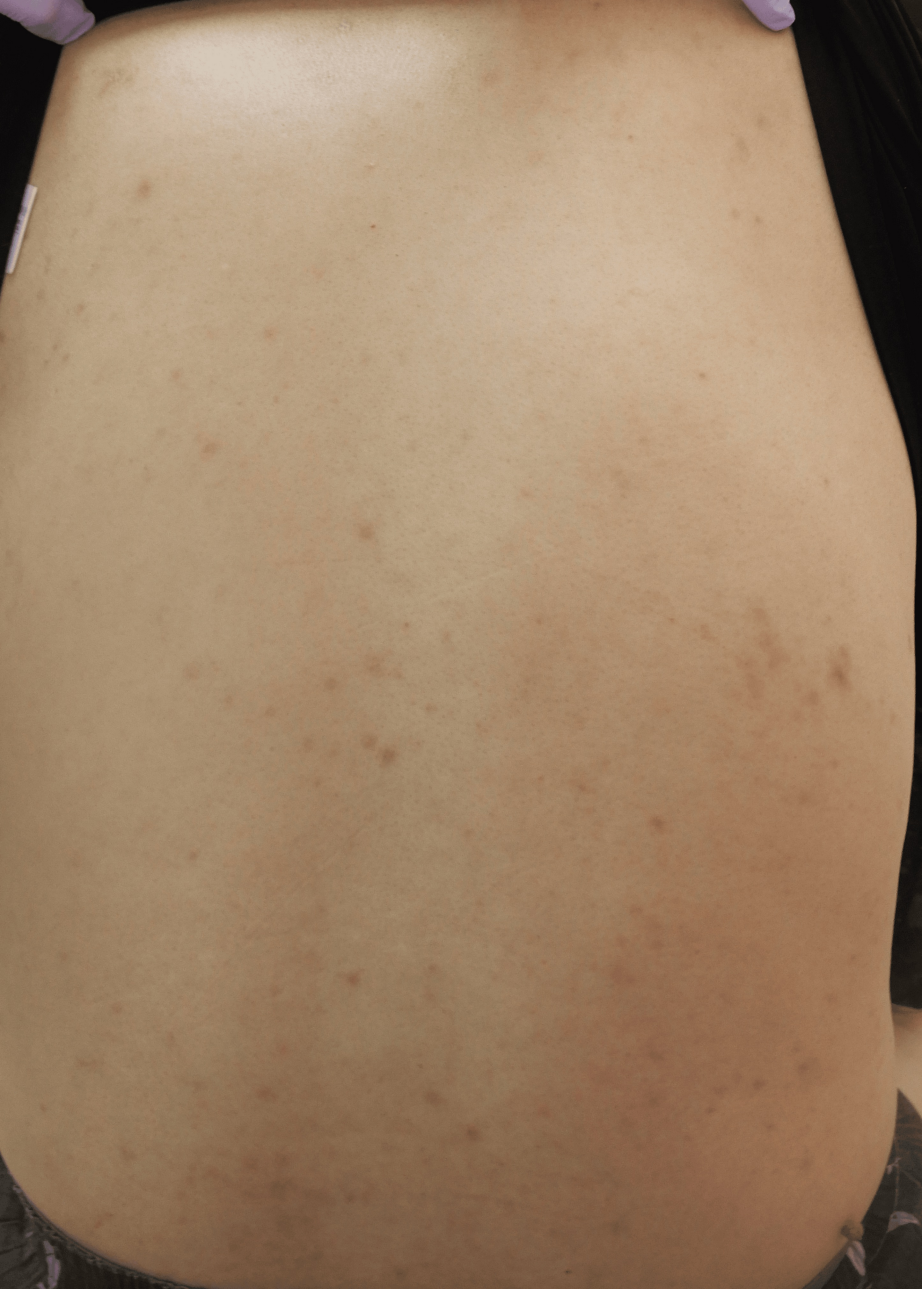
Lesions on the back show marked improvement at one week of topical steroid application.

Meanwhile, the trunk lesions largely resolved within one week, although the slower improvement in the right lower leg might have been influenced by the initial cellulitis (Figure 4).

The Baramycin® ointment was not reinitiated, and no recurrence of autoeczematization was observed.

## Discussion

Autoeczematization is an underrecognized complication arising from localized allergic skin inflammation [4]. In contact dermatitis, hapten-specific T-cell activation at the primary site can spread via the systemic circulation, resulting in secondary eczematous eruptions [4]. It is vital to recognize this phenomenon, as continued use of the offending agent or insufficient anti-inflammatory treatment may perpetuate or exacerbate the systemic reaction. Identifying and discontinuing the responsible agent, alongside initiating appropriate topical or systemic therapy, typically leads to rapid resolution of secondary lesions [5].

In addition to triggering autoeczematization, topical medicaments themselves frequently act as potent sensitizers [1]. Topical antibiotics are particularly associated with cosensitization, a phenomenon in which patients become sensitized to two chemically distinct allergens present in the same preparation [6]. For example, polymyxin B and bacitracin, which are often combined in over-the-counter antibiotic ointments, show considerable mutual cosensitization, and high rates of cosensitization have also been reported for neomycin, bacitracin, and polymyxin B [6]. Moreover, topical medicament ingredients can induce cross-reactions when structurally related allergens are encountered through different routes of exposure; in rare instances, such cross-reactions can lead to severe systemic reactions, including erythroderma or even anaphylaxis [6]. Thus, when prescribing topical or systemic antibiotics, it is important to obtain a careful history of previous drug or contact allergies and to consider possible cross-reactivity with related agents.

In treating patients who develop diffuse eczematous rashes following localized inflammation, especially when topical antibiotics or other sensitizers are used, clinicians should remain vigilant for autoeczematization. This case highlights the need to recognize autoeczematization as a complication of localized skin inflammation and to appreciate that allergy to topical antibiotics may indicate broader sensitization. Early suspicion in patients with widespread eczematous lesions enables timely intervention, more appropriate antibiotic selection, and better clinical outcomes.

## Conclusions

This report underscores the importance of considering autoeczematization in patients presenting with widespread erythema following the application of topical medicaments. Clinicians should inquire about recent topical antibiotic use, as these agents are common allergens and may trigger generalized eczematous reactions. Early recognition and withdrawal of the offending agent allow prompt resolution and prevent unnecessary investigations or continued exposure.
